# The role and mechanism of long non-coding RNA H19 in stem cell osteogenic differentiation

**DOI:** 10.1186/s10020-021-00350-y

**Published:** 2021-08-12

**Authors:** Liang Wang, Lei Qi

**Affiliations:** Department of Orthopaedic Surgery, Qilu Hospital of Shandong University, No.107, Wenhua Xi Road, Jinan, 250012 Shandong China

**Keywords:** lncRNA, H19, Stem cell, Osteogenic differentiation, Mechanism

## Abstract

**Background:**

In recent years, H19, as one of the most well-known long non-coding RNA, has been reported to play important roles in many biological and physiological processes. H19 has been identified to regulate the osteogenic differentiation of various stem cells in many studies. However, the detailed role and regulation mechanism of H19 was not consistent in the reported studies.

**Main body of the manuscript:**

In this review article we summarized the effect and mechanism of lncRNA H19 on osteogenic differentiation of various stem cells reported in the published literatures. The role and mechanism of H19, H19 expression changes, effect of H19 on cell proliferation in osteogenic differentiation were respectively reviewed.

**Conclusions:**

An increasing number of studies have provided evidence that H19 play its role in the regulation of stem cell osteogenic differentiation by different mechanisms. Most of the studies favored the positive regulatory effect of H19 through lncRNA-miRNA pathway. The function and underlying mechanisms by which H19 contributes to osteogenic differentiation require further investigation.

## Introduction

Long non-coding RNAs (lncRNAs) are a class of transcripts with sequence lengths of more than 200 nucleotides (Batista and Chang 2013; Kung et al. 2013). In recent years, mounting evidence has shown that various new bioinformatical and experimental strategies have identified a large number of novel lncRNAs (Lee and Bartolomei 2013; Batista and Chang, 2013; Alipoor et al. 2020). LncRNAs could regulate gene expressions through interactions with DNAs, RNAs, protein (Koch 2017; Ali and Grote 2020; Cardon et al. 2020; Li et al. 2020a, b; Zhang et al. 2021), including chromatin remodeling, as well as transcriptional, post-transcriptional and epigenetic regulations (Lee and Bartolomei, 2013; Fatica and Bozzoni, 2014; Graf and Kretz 2020; Li et al. 2020a, b).

LncRNA H19, one of the most well-known imprinted genes, was firstly isolated and reported in 1980s, and is located on human chromosome 11p15.5 (Zhang and Tycko 1992; Hurst and Smith 1999; Cai and Cullen 2007). H19 is transcribed only from the maternally inherited allele and it does not encode protein, but rather a 2.3-kb H19 ncRNA (Zhang and Tycko 1992). During the early stages of embryogenesis in humans, H19 gene in expressed mainly in the adrenal, muscle, and liver (Goshen et al. 1993). Among the adult human tissues, H19 was significantly decreased and mainly expressed in skeletal muscle and heart (Gabory et al. 2010). As an imprinted gene, H19 performs a valuable biological function with a very low mutation rate in exons (Hurst and Smith 1999). H19 is highly expressed during fetal development, and dramatically reduced in adult tissues after birth, indicating its highly conserved characteristic throughout evolution and it has an important biological function (Ayesh et al. 2002; Goodell 2013).

As one of the most well-known lncRNAs, H19 has been implicated in human disorders through various molecular mechanisms, including controlling of RNA progressing, cellular proliferation, differentiation, and disease development (Ratajczak 2012). The diagnostic and therapeutic importance of H19 in human cancer has been widely established (Alipoor et al. 2020; Shermane et al. 2021)**,** such as breast cancer (Li et al. 2020a, b), lung cancer (Xu et al. 2019)**.** H19 was also shown to play an important role in various cardiovascular diseases such as acute myocardial infarction (Huang et al. 2020), myocardial I/RI (Li et al. 2019a, b) and cardiomyocyte hypertrophy (Viereck et al. 2020). It has also been confirmed that H19 plays a noticeable role in embryonic placental growth, skeletal muscle differentiation and related diseases (Dey et al. 2014; Zhang et al. 2020).

Stem cell osteogenic differentiation is a key stage and complex process in bone formation involving many genes and signaling pathways (Nancarrow-Lei et al. 2017; Ju et al. 2019; Halim et al. 2020). It has been widely proved that H19 participates and plays an important role in osteogenic differentiation of various stem cells (Peng et al. 2018). However, the exact regulatory functions and mechanisms of H19 remain to be elucidated.

## The role and mechanism of H19 in osteogenic differentiation

**Fig. 1:**
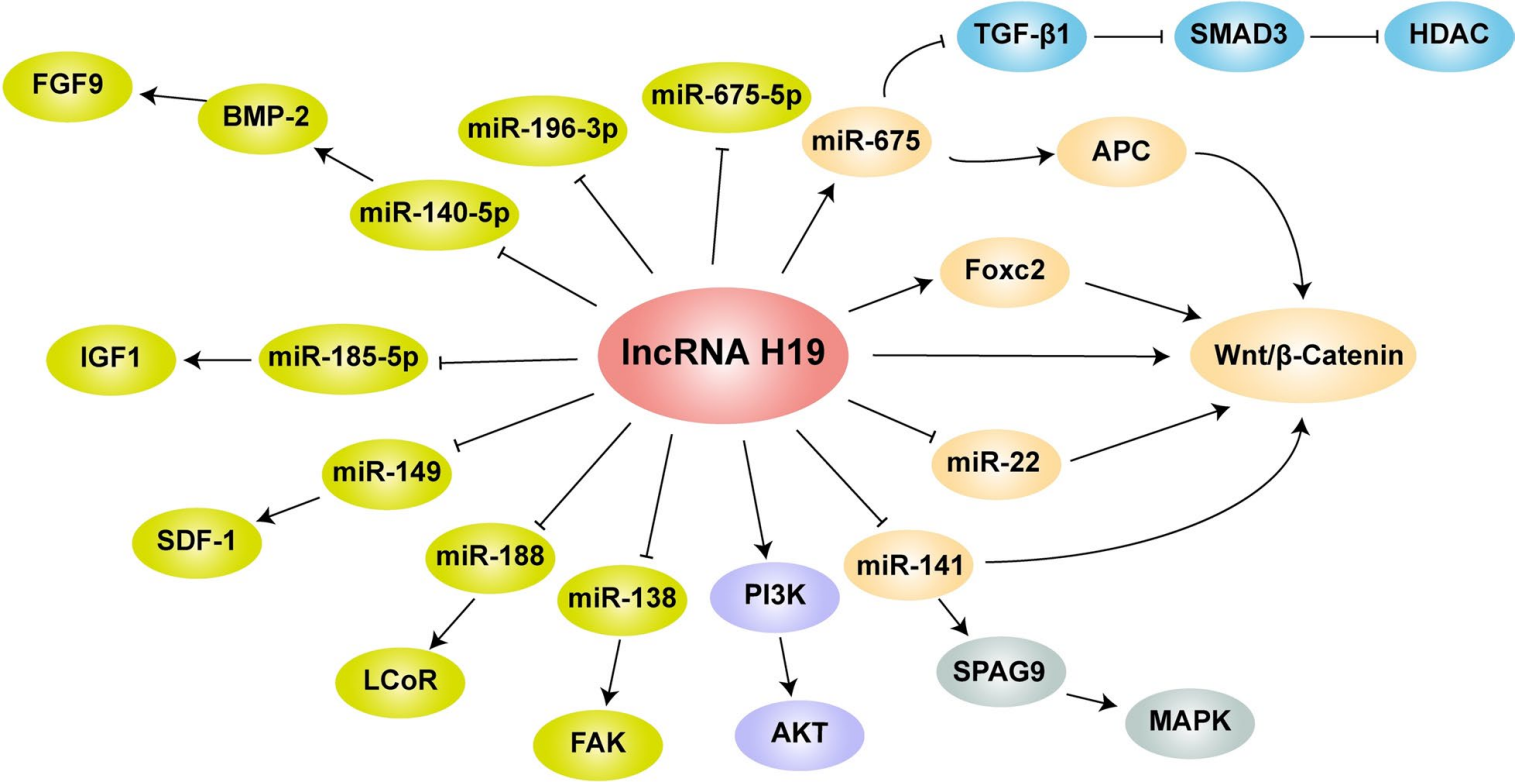
The pathways and mechanisms of lncRNA H19 promoting osteogenic differentiation reported in previous published studies

There were eighteen studies were identified in the current review (Table 1). As for the positive or negative effect of H19 on the osteogenic differentiation, there were also some inconsistencies in the observed results. The pathways and mechanisms of lncRNA H19 promoting osteogenic differentiation reported in previous published studies were shown in Fig. 1. Sixteen out of all eighteen studies demonstrated the positive effect of H19 on the osteogenic differentiation.

**Table 1:** Eighteen studies reporting H19 in osteogenic differentiation identified in the current review

	Role of H19	Experimental cells	Mechanism or pathway of H19
Huang et al. (2015)	Positive	hMSCs	H19/miR-675/TGF-β1/Smad3/HDAC
Ma et al. (2020)		hBMSCs	H19/miR-675/APC/Wnt/β-catenin
Liang et al. (2016)		hMSCs	H19/miR-141/Wnt/β-catenin H19/miR-22/Wnt/β-catenin H19/miR-675-5p
Li et al. (2019a, b)		SCAPs	H19/miR-141/SPAG9/MAPK
Wu et al. (2018)		hBMSCs	H19/miR-138/FAK
Wang et al. (2018)		Mouse BMSCs	H19/miR-188/LCoR
Wu et al. (2019)		Mouse osteoblasts	H19/miR-185-5p/IGF1
Zhong et al. (2020)		hDPSCs	H19/miR-140-5p/BMP-2/FGF9
Li et al. (2020a, b)		Rat BMSCs	H19/miR-149/SDF-1
Xiaoling et al. (2020)		hMSCs	H19/miR-19b-3p
Gong et al. (2018)		Rat ectomesenchymal stem cells	H19/Wnt/β-catenin
Zhou et al. (2019)		Mouse BMSCs	H19/Foxc2/Wnt/β-catenin
Xie et al. (2019)		Rat MSCs	H19/PI3K/AKT H19/Wnt/β-catenin
Zhu et al. (2020)		Renal interstitial fibroblasts	H19/Wnt/β-catenin
Izadpanahi et al. (2018)		hASCs	–
Liao et al. (2020)		Mouse MSCs	–
Huang et al. (2017)	Negative	hASCs	–
Liao et al. (2017)	Biphasic	Mouse MSCs	–

*hMSCs* human mesenchymal stem cells, *BMSCs* bone marrow mesenchymal stem cells, *hASCs* human adipose-derived stem cells, *SCAPs* stem cells from apical papilla, *hDPSCs* human dental pulp stem cells

There were three studies revealed the H19/miR-675 coordination effect. Huang et al. demonstrated that the novel pathway H19/miR-675/TGF-β1/Smad3/HDAC regulates osteogenic differentiation of human mesenchymal stem cells (hMSCs) and H19 promotes bone formation in vivo (Huang et al. 2015). H19 and encoded miR-675 were significantly upregulated after the induction of osteogenic differentiation. H9/miR-675 inhibited mRNA and protein expression of TGF-β1. The downregulation of TGF-β1 subsequently inhibited phosphorylation of Smad3. Meanwhile, H19/miR-675 downregulated the mRNA and protein levels of HDAC4/5, and thus increased osteogenic marker gene expression. Ma et al. revealed that human amnion-derived MSCs (hAMSCs) promote osteogenic differentiation of human bone marrow mesenchymal stem cells (hBMSCs) via H19/miR-675/APC/Wnt/β-catenin axis (Ma et al. 2020). H19 promoted miR-675 expression and contributed to the competitively bounding of miR-675 and APC, thus significantly activating the Wnt/β-catenin pathway. Liang et al. revealed that H19 promotes osteogenic differentiation of hMSCs with the pathway of H19/miR-675-5p (Liang et al. 2016). H19 is negatively regulated by miR-675-5p, which alleviates osteogenic differentiation and was found to directly target H19 and counteracted differentiation.

There were two studies focused on H19/miR-141 axis. Liang et al. revealed that H19 promotes osteogenic differentiation of hMSCs with the pathway of H19/miR-141/ Wnt/β-catenin (Liang et al. 2016). H19 was found to be upregulated during osteogenic differentiation in hMSCs and functioned as a ceRNA sponging for miR-141, which was negative regulator of osteogenesis and Wnt/β-catenin pathway. Li et al. revealed that H19 promotes the committed differentiation of stem cells from apical papilla (SCAPs) and “H19/miR-141/SPAG9/MAPK” positive feedback loop plays paramount role (Li et al. 2019a, b). Mechanistically, H19 competitively bound to miR-141 and prevented SPAG9 from miRNA-mediated degradation, thus significantly elevating phosphorylated levels of p38 and JNK and facilitating the committed differentiation of SCAPs. H19, as a ceRNA, serves as a miRNA sponge for miR-141.

There were other miRNAs were found to participate in the process of H19 regulating the osteogenic differentiation, including miR-22, miR-138, miR-188, miR-185-5p, miR-140-5p, miR-149, miR-19b-3p. Liang et al. revealed that H19 promotes osteogenic differentiation of hMSCs with the pathway of H19/miR-22/Wnt/β-catenin (Liang et al. 2016). Wu et al. revealed that H19 mediates mechanical tension-induced osteogenesis of hBMSCs via H19/miR-138/FAK pathway (Wu et al. 2018). Mechanical tension could suppress miR-138 expression, and down-regulated miR-138 promoted tension-induced osteogenesis. H19, as a ceRNA, had binding sites with miR-138, and overexpression of H19 decreased the level of miR-138, then targeted PTK2 and up-regulating downstream FAK. Wang et al. revealed the regulatory effect of H19/miR-188/LCoR axis on the osteogenic and adipogenic differentiation of mouse BMSCs (Wang et al. 2018). H19 mediated LCoR to regulated the balance between osteogenic and adipogenic differentiation of BMSCs in mice through sponging miR-188. The expression of miR-188 was lower and H19 was higher in osteogenesis induced mouse BMSCs. Meanwhile, H19 and LCoR were downregulated in adipogenic induced mouse BMSCs. Wu et al. showed that H19/miR-185-5p/IGF1 axis in modulating matrix mineralization in mouse MC3T3-E1 osteoblasts for the first time (Wu et al. 2019). H19 and IGF1 were highly expressed while miR-185-5p was lowly expressed in mineralized cells. H19 acts as a ceRNA by sponging miR-185-5p and regulated IGF1 expression indirectly. Zhong et al. found that H19 plays a positive regulatory role in odontoblastic differentiation of human dental pulp stem cells (hDPSCs) through H19/miR-140-5p/BMP-2/FGF9 axis (Zhong et al. 2020). The expression of H19 was significantly upregulated and overexpression of H19 stimulated odontoblastic differentiation in vitro and in vivo. H19, as a ceRNA, acted as a miR-140-5p sponge, resulting in regulated the expression of FGF9. Li et al. found that H19 stimulates osteogenic differentiation of rat BMSCs via the H19/miR-149/SDF-1 axis (Li et al. 2020a, b). Overexpressed H19 and SDF-1 and poorly expressed miR-149 were found in rats with osteogenic differentiation. H19 enhanced ALP activity, OCN content, calcium deposit and ALP, OCN, RUNX2 and OSX protein expression of BMSCS by up-regulating SDF-1 via binding to miR-149. Xiaoling et al. firstly revealed the critical role of H19/miR-19b-3p in postmenopausal osteoporosis and osteogenic differentiation of hBMSCs (Xiaoling et al. 2020). The significant decrease of H19 and increase expression of miR-19b-3p were found in postmenopausal osteoporosis patients. H19 up-regulation elevates cell proliferation and differentiation of hBMSCs through mediating miR-19b-3p.

Wnt/β-catenin signaling pathway has been proved to be an important regulator during the osteogenic differentiation. There were five studies reported the Wnt/β-catenin involved in the regulation process of H19. Ma et al. and Liang et al. respectively revealed the H19/miR-675/APC/Wnt/β-catenin (Ma et al. 2020) and H19/miR-141/miR-22/Wnt/β-catenin axis (Liang et al. 2016) in osteogenic differentiation of hMSCs. Gong et al. found that H19 promotes the osteogenic differentiation of rat ectomesenchymal stem cells via H19/Wnt/β-catenin signaling pathway (Gong et al. 2018). Down-regulation of H19 repressed expression of ALP, Runx2, BMP, OCN, β-catenin, c-myc and CD44. H19 activated Wnt/β-catenin signaling by inhibiting the effect of miR-141 and miR-22. Zhou et al. reported that H19 and Foxc2 synergistically promotes osteogenic differentiation of mouse BMSCs via Wnt/β-catenin pathway (Zhou et al. 2019). H19 expression was reduced in the serum of patients with postmenopausal osteoporosis and BMSCs of ovariectomized mice. Overexpression of H19 promoted osteogenic differentiation of BMSCs. H19 could bind to Foxc2 and H19/Foxc2/Wnt/β-catenin pathway maybe the key mechanism. Xie et al. found that angelica polysaccharide promotes rat MSCs osteogenic differentiation by regulating H19 (Xie et al. 2019). angelica polysaccharide could upregulate the expression level of H19 in MSCs and promoted the activation of PI3K/AKT and Wnt/β-catenin signaling pathways. Zhu et al. found that H19 promotes osteogenic differentiation of renal interstitial fibroblasts through Wnt/β-catenin pathway (Zhu et al. 2020).

There were other studies also reported the positive effect of H19 on osteogenic differentiation but without the detailed mechanism described in the article. Izadpanahi et al. demonstrated the H19 modulation to osteogenic differentiation of human adipose tissue-derived mesenchymal stem cells (hASCs) during BMP signaling pathway (Izadpanahi et al. 2018). The expression of H19 was significantly increased from day 7 and maintained at a high level at day 21. Liao et al. revealed that exogenous expression of H19 biphasic regulating osteogenic differentiation of mouse MSCs (Liao et al. 2020). Higher dosage of H19 inhibited and lower H19 promoted osteogenic differentiation.

There was just one study manifesting the negative effect of H19 on the osteogenic differentiation. Huang et al. reported that H19 expression decreased (Fold change: 3.81) significantly during osteogenic differentiation of hASCs (Huang et al. 2017). Silencing of H19 caused a significantly increase in expression of osteogenesis-related genes, including ALP and Runx2.

In addition, there was one study demonstrating the biphasic regulation of H19. Liao et al. reported that H19 mediates BMP9-induced osteogenic differentiation of mouse MSCs through Notch signaling (Liao et al. 2017). Both overexpression and silencing of H19 inhibit the terminal differentiation of BMP9-induced ectopic bone formation from MSCs. H19 may play a delicate role in fine-tune regulation of BMP9-induced osteogenic differentiation of MSCs.

## H19 expression changes in osteogenic differentiation

Despite the roles of H19 in osteogenic differentiation has been widely analyzed and reported, the change of H19 expression level has just been reported in several studies. Ma et al. found that RNA samples derived from hAMSCs expressed significantly increased levels of H19 in a time-dependent manner along with the osteogenic differentiation of hBMSCs (Ma et al. 2020). Liao et al. firstly explored the expression level of H19 in different time points of osteogenic differentiation of mouse MSCs (Liao et al. 2017, 2020). The study revealed that H19 expression level increased gradually from day 1 to day 3, and reach the peak at day 3. Then, the expression of H19 decreased gradually and maintained in a relatively high level at day 7 and day 9. Izadpanahi et al. demonstrated the expression of H19 was significantly increased from day 7 and maintained at a high level at day 21 during the osteogenic differentiation of hASCs (Izadpanahi et al. 2018).

## Effect of H19 on cell proliferation in osteogenic differentiation

It has been widely proved that H19 plays important roles in osteogenic differentiation of MSCs. As for the effect of H19 on the proliferation of MSCs, there were just several studies but with different results. There were three researches revealed that H19 had no significant effect on the proliferative behaviors of hMSCs (Liang et al. 2016; Ma et al. 2020) and SCAPs (Li et al. 2019a, b). Meanwhile, Zhao et al. found that DLX3 promotes hBMSCs proliferation through H19/miR-675 axis (Zhao et al. 2017). Xiaoling et al. found that H19 up-regulation elevates cell proliferation hBMSCs through mediating miR-19b-3p (Xiaoling et al. 2020).

## Conclusions

In recent years, lncRNA H19 have received widespread attention as emerging regulators of stem cell differentiation, especially the osteogenic differentiation. An increasing number of studies have provided evidence that H19 plays its role in the regulation of osteogenic differentiation by different mechanisms and most of the studies favored the positive regulatory effect of H19. Despite various mechanisms reported in previous published studies, most of the studies focused on the H19-miRNA interactions but with different pathways and the results in some studies was just superficial phenomenal needing in-depth investigation. Even though our current understanding of its function is continuously expanding, existing knowledge gaps need to be addressed in the future, especially for the underlying mechanisms involving lncRNA-miRNA pathways.

## Data Availability

Not applicable.

## Abbreviations

lncRNA: Long non-coding RNA
hMSCs: Human mesenchymal stem cells
hAMSCs: Human amnion-derived MSCs
hBMSCs: Human bone marrow mesenchymal stem cells
SCAPs: Stem cells from apical papilla
hDPSCs: Human dental pulp stem cells
hASCs: Human adipose tissue-derived mesenchymal stem cells
